# Hypereosinophilia in childhood acute lymphoblastic leukaemia at diagnosis: report of 2 cases and review of the literature

**DOI:** 10.1186/1824-7288-40-36

**Published:** 2014-04-10

**Authors:** Rosanna Parasole, Fara Petruzziello, Antonia De Matteo, Giovanna Maisto, Luisa Castelli, Maria Elena Errico, Giuseppe Menna, Vincenzo Poggi

**Affiliations:** 1 Department of Paediatric Haemato-Oncology, Santobono-Pausilipon Children’s Hospital, Via Posillipo, 226, 80123 Napoli, Italy; 2 Service of Diagnostic Radiology, Santobono-Pausilipon Children’s Hospital, Napoli, Italy; 3 Service of Pathology, Santobono-Pausilipon Children’s Hospital, Napoli, Italy

**Keywords:** Hypereosinophilia, Acute lymphoblastic leukaemia, Childhood

## Abstract

Hypereosinophilia as first clinical presentation has rarely been reported in paediatric acute lymphoblastic leukaemia. It is commonly associated with specific cytogenetic abnormalities. Although eosinophilia is considered a reactive, non-neoplastic epiphenomenon, it adversely affects patient outcomes, both in children and adults. We describe herewith two paediatric patients who had marked eosinophilia at onset of acute lymphoblastic leukaemia. We point out the importance of a correct differential diagnosis in persistent, unexplained peripheral hypereosinophilia. Clinicians should keep in mind that eosinophilia can be part of the overall pattern of acute leukaemia and therefore needs to be properly investigated. We also provide some recommendations for an appropriate approach to hypereosinophilia - related morbidities.

## Background

Hypereosinophilia is frequently documented with parasitic infections, allergic disorders, and some haematologic malignancies, including acute lymphoblastic and chronic myeloid leukaemias, and Hodgkin’s lymphoma [1].

The association of acute lymphoblastic leukaemia (ALL) and hypereosinophilia represents a distinct clinico-pathological entity [2-4], although rarely described in childhood [2,5,6], with 44 paediatric cases reported to date [7]. In these patients, presenting symptoms are non-specific and include intermittent low-grade fever, fatigue and “purpuric” rash [8]. Although hypereosinophilia has commonly been interpreted as a reactive epiphenomenon rather than the result of a leukemic clone [9], its occurrence appears to be associated with worse prognosis, both in children [6] and adults [10]. Cytogenetic abnormalities, such as t(5;14), are commonly associated with this subtype of leukaemia [9].

We report herewith two paediatric cases of eosinophilia at diagnosis of acute lymphoblastic leukaemia, presenting to our institution almost simultaneously, thus raising the question of a possible common etiology.

## Case presentations

### Case 1. B.V

A 13-year-old girl was referred in June 2011 to our Institution for persistent low-grade fever and fatigue. On admission, physical examination was negative except for noticeable swelling of the inferior left leg, that Doppler ultrasound documented to be secondary to deep venous thrombosis of the left iliac-femoral axis. Laboratory findings showed a normal leukocyte count (WBC 7,870/μL) with marked hypereosinophilia (44.3%), mild anemia (Hb 9.8 g/dL) and thrombocytopenia (129,000/μL). Peripheral blood smears revealed mature eosinophils and no abnormal cells. Main parasitic infections and allergic conditions were ruled out. Bone marrow aspirates showed a prevalence of FAB L2 lymphoblasts (52%) associated with 30% of eosinophilic elements. Immunophenotyping revealed CD10-positive blasts of B-lymphoid lineage, with cytogenetic analysis showing normal 46, XX karyotype. Molecular diagnostic studies detected clonal rearrangement of the immunoglobulin heavy-chain (IgH) region, while the main molecular leukaemic rearrangements, including bcr/abl, TEL/AML1, MLL/AF4, E2A-Pbx1, 4q *FIP1L1/PDGFRA* fusion genes, turned negative [11].

Patient was enrolled in the AIEOP-BFM ALL 2000 protocol [12], excluding L-Asparaginase (L-ASP), given the concomitant venous thrombosis for which enoxaparin was instituted. Following the 8-day prednisone pre-phase therapy, marked drop of peripheral eosinophil count was evident. At day 15, bone marrow was free of blast cells. Thrombophilic profile documented genetic predisposition to thrombosis consisting of homozygous for -455G/A of b-fibrinogen (Table 1). At day 78, patient was assigned to intermediate risk for minimal residual disease, measured by real-time quantitative polymerase chain reaction (PCR) [13]. At the end of induction, the thrombotic pattern had disappeared, but enoxaparin prophylaxis was continued until maintenance. Consolidation therapy was well tolerated and during re-induction therapy, L-ASP was administered without any adverse effects. Presently, at 7 months from end of therapy and 31 months from diagnosis, patient is in complete continuous remission.

**Table 1:** Patients’ clinical features and laboratory results

**Characteristics**	**Patient #1**	**Patient #2**
**Sex**	Female	Male
**Age at diagnosis**	13 years	20 months
**Peripheral blood:**		
*Hb (g/dL)*	9.8	12
*White blood cells count (μL)*	7,870	14,390
*Eosinophils count (μL)(%)*	3,490 (44.3%)	4,620 (32.1%)
*Platelets count (μL)*	129,000	223,000
*Blasts (%)*	None	2
**Bone marrow aspirate:**		
*Blasts (%)*	52	60
*Eosinophils (%)*	30	36
**FAB classification**	L2	L1
**Immunophenotype**	Common	Common
**DNA index**	1	1,1
**Bone marrow biopsy**	B cells lymphoproliferative syndrome; MF1 (WHO grading)	B cells lymphoproliferative syndrome; MF0 (WHO grading)
**Karyotype**	46, XX	Hyperdiploid (52,XXY,+6,+14,+17,+21,+21[5])
**Molecular biology for ALL rearrangements**	Negative	Negative
**Molecular biology for thrombophilia**	Positive (Homozygous for -455G/A of b-fibrinogen)	Positive (Eterozygous for P1b of HPA)
**Clinical presentation**	Evening fever	Night sweat
	Left iliac-femoral deep venous thrombosis	Fever
		Pruritic erythroderma
		Claudication for left inferior limb arthralgia
**Other organ involvement**	No	Skin
		Bone (tibial osteolysis)

FAB, French-American-British classification; MF, myelofibrosis; WHO, world health organization; HPA, human platelet antigen; ALL, acute lymphoblastic leukaemia.

### Case 2. R.S

**Figure 1:**
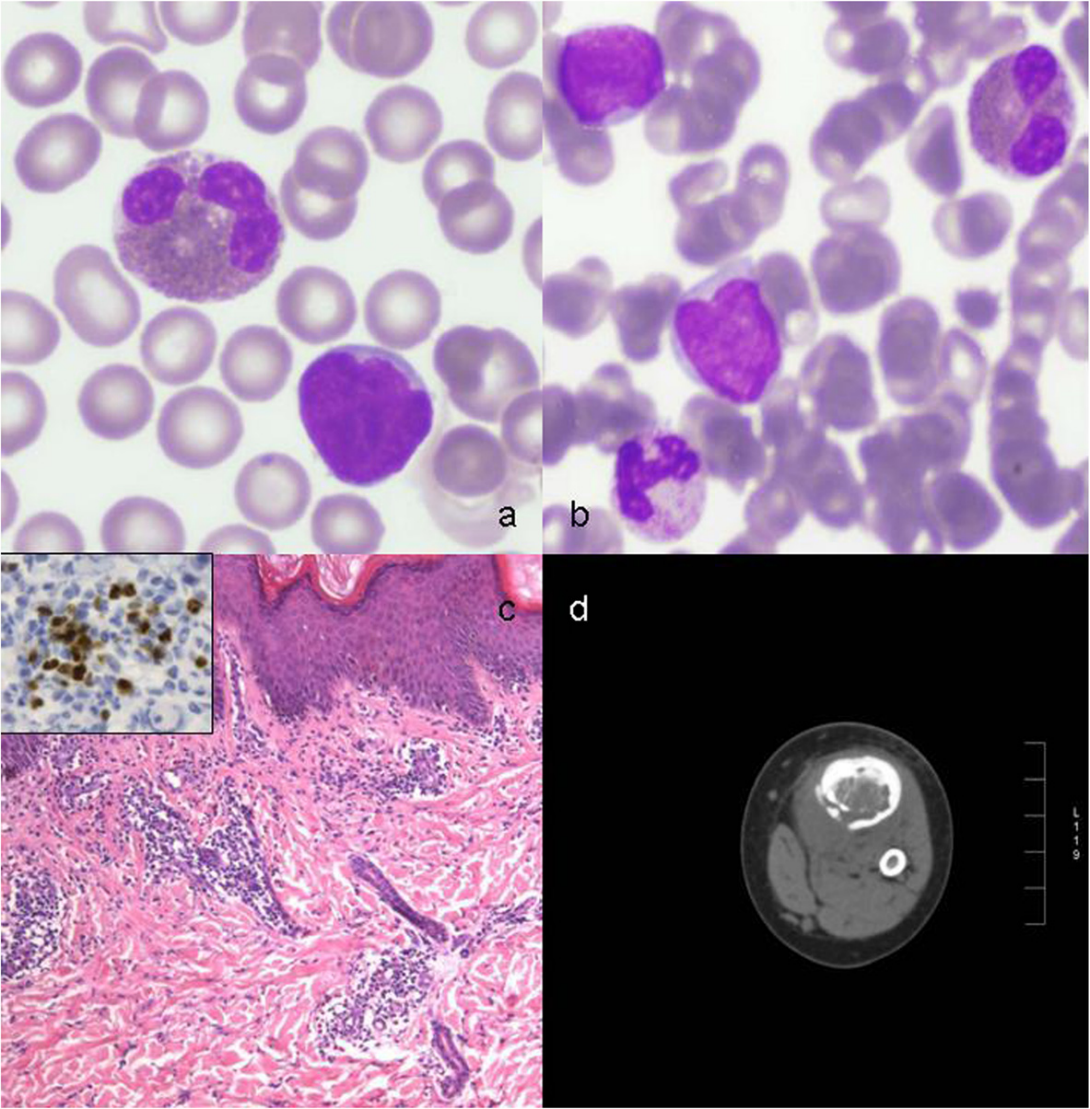
**Patients’ hematopathology and radiology. a)** Peripheral smear showing lymphoblasts and eosinophils (May-Grünwald -Giemsa); **b)** Bone marrow smear showing FAB L1-lymphoblasts, eosinophils and neutrophils (May-Grünwald - Giemsa); **c)** Skin biopsy showing perivascular infiltration of small lymphocytes and eosinophils (Hematoxylin-eosin, original magnification × 100); Inset: lymphocyte’s nuclear TdT positivity in immunohistochemistry (ABC staining, × 400); **d)** CT scan demonstrating tibial osteolytic area with interruption of cortical bone profile.

In July 2011, fifteen days after the previous patient’s admission, a 20 month-old boy with recent history of viral illness (hand-foot-mouth disease), followed by the appearance of an erythematous papular rash (pruritic erythroderma), fever, night sweats and claudication for left inferior limb arthralgia, was referred to us for consultation. Blood count showed mildly increased white blood cell count (WBC 14,390/μL) with hypereosinophilia (4,620/μL). Peripheral blood smears showed marked hypereosinophilia with 2% of immature cells (Figure 1a). Laboratory investigations were negative for parasitic infections and allergic diseases. Bone marrow aspirates revealed a prevalence (60%) of L1 CALLA positive lymphoblasts with 36% eosinophils (36%) (Figure 1b). The histologic examination of a cutaneous lesion showed infiltration by leukaemic cells associated with eosinophilia (Figure 1c). Computerized tomography (CT) of left inferior limb documented a tibial osteolytic area with interruption of cortical bone profile (Figure 1d); histologically, the lesion revealed leukaemic and eosinophilic infiltration. Cytogenetic studies showed a hyperdiploid karyotype (52 chromosomes), while the main leukaemic rearrangements, including bcr/abl, Jak2 and 4q(12) [11] were negative (Table 1). Treatment with the AIEOP-BFM ALL 2000 protocol [12] was initiated, leading to prompt response to prednisone on day 8. On day 15, patient was in complete haematological remission. To prevent hypereosinophilia-related thrombotic complications, prophylactic enoxaparin was administered until the end of the induction phase, with transient interruptions when platelet count lowered to < 30,000/μL. The thrombophylic profile showed genetic predisposition to thrombosis (Table 1). Following results of PCR that showed negativity of both markers at day 33 and 78, patient was assigned to standard-risk [13]. Consolidation and re-induction phases were performed without complications. Presently, patient remains in full continuous remission, six months after conclusion of maintenance therapy. Patient’s clinical features and laboratory results are listed in Table 1.

## Discussion and conclusions

Less than 1% of children with ALL present with hypereosinophilia at time of diagnosis [14]. These cases are prevalently male patients, median age at presentation of 14 years (range, 2–58). The ALL is mostly characterized by a B-lineage phenotype [15-17]. Eosinophilia generally precedes the diagnosis of ALL by 1 to 9 months and quickly resolves with achievement of disease remission, but tends to accompany relapse, if it occurs. Although several hypotheses have been proposed [16], whether this reappearance is due to leukaemia or to an associated infection remains unclear.

In this report, we have described 2 pediatric cases, presenting to us within a 15 day interval, with eosinophilia as first clinical presentation of ALL. Of note, in the series of 341 ALL patients diagnosed in our institution and enrolled in the ALL-2000 protocol in a 10-year period, only these 2 patients had hypereosinophilia at onset. Our findings might suggest that eosinophilia is a reactive condition to some pathogen, such as seasonal infection. In fact, patient # 2 had a positive history for recent viral illness (hand-foot-mouth disease). Other investigators have demonstrated the non-clonal origin of eosinophils in ALL [1-6,9,10], but further cytogenetic and molecular studies are required to confirm these observations, given the rarity of this association. A possible hypothesis to explain the occurrence of hypereosinophilia could be the production by leukemic cells of stimulating eosinophilic factors, such as interleukin 3 (IL-3) or IL-5 [9,14]. Other exogenous factors, *ie* viral infections, could contribute to cytokine production with subsequent hypereosinophilia.

Some cytogenetic abnormalities, frequently reported in precursor B-acute lymphoblastic leukaemia associated with marked eosinophilia, [1,4,9] could be involved in the complex mechanism of leukaemogenesis, [9] or in over-expression of eosinophil growth factors. The most common cytogenetic findings involve chromosomes 5 and 14, t(5;14) (q31;q32) and chromosome 5 deletion, del(5)(q15q33). Of interest, chromosome 5 carries IL-3 gene, whose overexpression leads to increased eosinophil production [14]. Cytogenetic abnormalities were found only in leukaemic blasts while eosinophils presented normal karyotype in 90% of the cases [9]. Nevertheless, the relationship between eosinophilia and leukaemia remain controversial. In the two patients reported herein, no characteristic chromosomal abnormalities were observed, with hyperdiploidy in patient # 2, not involving chromosome 5.

The persistence of peripheral hypereosinophilia should still lead the clinician to exclude an idiopathic hypereosinophilic syndrome (HES), a clonal proliferation secondary to a myeloproliferative disorder with obvious prognostic and therapeutic implications [18]. Therefore, the presence of specifically molecular genetic abnormality *FIP1L1–PDGFRA* fusion gene, is in favour of the hypothesis that this haematological disorder represents a chronic eosinophilic leukaemia. In case a persistent eosinophilia is documented, diagnosis of HES must ruled out. Our patients were both negative for 4q(12) rearrangement, as well as for Jack2 and brc/abl.

Prognosis of ALL presenting with hypereosinophilia is definitely worse both in children [6] and adults [10], with median survival of 7.5 months [15]. With the limitations of a short follow-up (30-months median survival), both our patients are in haematological and molecular continuous complete remission.

In patients bearing this association, a significantly increased risk of cardiac and vascular thrombosis exists [19]. Congestive heart failure represents the main cause of mortality in patients with ALL and hypereosinophilia [15,19]. We observed a thrombotic complication at diagnosis in patient # 1, successfully resolved with anticoagulant therapy. Clinicians should therefore keep in mind that thrombotic complications may occur in this ALL subset in order to plan prompt heparin prophylaxis or chemotherapic modulation of prothrombotic drugs, such as L-ASP.

Pruritic erythroderma represents an additional common presenting feature in children with ALL and hypereosinophilia [9]. In this regard, patient # 2 showed a diffuse itchy erythematous-papular erythroderma at onset, misdiagnosed even by expert dermatologists. Therefore, skin biopsy is a mandatory procedure in case of cutaneous lesions of difficult differential diagnosis in order to confirm a possible infiltration by malignant cells (Figure 1c).

In conclusion, the poor prognosis reported for these atypical ALL should prompt clinicians to carry out accurate follow-up including peripheral eosinophil count that could be the first manifestation of relapsed leukaemia. In addition, this unique ALL form presents additional morbidity and mortality related to hypereosinophilia, such as cardiac failure, peripheral neuropathy and thromboembolic phenomena. Follow-up should therefore include careful surveillance of secondary complications.

Based on our experience, we suggest that paediatricians and haematologists should be aware of this unusual presentation of ALL within the context of a correct differential diagnosis of persistent peripheral eosinophilia, particularly in the absence of lymphoblasts in peripheral blood. We point out that persistent hypereosinophilia may occur together with ALL also in the absence of anemia, thrombocytopenia and leukocytosis, thus requiring appropriate investigation, including bone marrow aspirate [20].

## Consent

Written informed consent was obtained from patients’ parents for publication of these Case reports and any accompanying images. A copy of the consent form is available for appraisal by the Editor-in-Chief.

## Abbreviations

ALL: Acute lymphoblastic leukaemia; CD: Cluster of differentiation; PCR: Polymerase chain reaction; AIEOP: Associazione Italiana di Ematologia ed Oncologia Pediatrica; BFM: Berlin-Frankfurt-Muenster; FAB: French-American-British; L-ASP: L-asparaginase; IL: Interleukin; CT: Computerized tomography; ABC: Avidin Biotin Complex.

## Competing interests

The authors declare no conflicts of interest.

## Authors’ contributions

RP, FP, ADM contributed to data collection and interpretations, and to draft the paper; GM, VP revised critically the manuscript; GM, LC, MEE contributed to diagnostic investigations and images. All authors read and approved the final version of the manuscript.
